# Assessing Individual Change Without Knowing the Test Properties: Item Bootstrapping

**DOI:** 10.3389/fpsyg.2018.00223

**Published:** 2018-03-13

**Authors:** Juan Botella, Desirée Blázquez, Manuel Suero, James F. Juola

**Affiliations:** Department of Social Psychology and Methodology, Universidad Autónoma de Madrid, Madrid, Spain

**Keywords:** bootstrap, individual change, reliable change, significant change, psychometric properties, meta-analysis

## Abstract

Assessing significant change (or reliable change) in a person often involve comparing the responses of that person in two administrations of a test or scale. Several procedures have been proposed to determine if a difference between two observed scores is statistically significant or rather is within the range of mere random fluctuations due to measurement error. Application of those procedures involve some knowledge of the test properties. But sometimes those procedures cannot be employed because the properties are unknown or are not trustworthy. In this paper we propose the *bootstrap of items* procedure to create confidence intervals of the individual's scores without using any known psychometric properties of the test. Six databases containing the responses of several groups to one or more subscales have been analyzed using two methods: bootstrap of items and a classical procedure based on confidence intervals to estimate the true score. The rates of significant change obtained were very similar, suggesting that item bootstrapping is a promising solution when other methods cannot be applied.

## Introduction

Often in disciplines like psychology or education it is important to be able to measure a change in a person's behavior. Such change can be the result of an intervention or of a natural development. When the change reflects a construct that can be measured by a scale or test, the procedure essentially consists of administering that test before and after an intervention and comparing the results. In clinical and health settings, a distinction is usually made between the presence of some change against the situation in which there is no change (i.e., a *significant or reliable change*) or the presence of a change whose magnitude reaches a certain level against smaller amounts of change (*clinically significant change*) (Jacobson and Truax, 1991; Kazdin, 1999, 2001; Kendall, 1999; Ogles et al., 2001; Perdices, 2005). The first type of change is defined by an arithmetic difference between two successive observations that may or may not be due to random fluctuations. That is, whether or not the observed difference exceeds the range of differences that can reasonably be expected from mere oscillations due to the measurement error. If the observed difference falls outside of a predefined range of measurement errors, then it is concluded that the change is statistically significant or that a reliable change has occurred. The second type of change concerns whether, in addition to being reliable, the change reaches a magnitude that is significant for diagnostic decisions or interventions. That is, if the observed change reaches a certain level, relevant according to some criterion. In this paper our focus is on the first one, the statistically significant change.

A significant or reliable change can also be observed for reasons other than a genuine change in the variable of interest. For example, a mere mistake in typing or calculating one or both scores can also yield statistically different scores. They can also be observed because in fact two different persons participate in the before and after tests, in cases of fraud by impersonation. Detecting significant differences when they should not appear can help to identify false positives due to several sources, beyond the expected rate given the statistical nature of the procedure.

Several methods have been proposed to determine whether an observed difference reliably reflects a change. Ferrer and Pardo (Pardo and Ferrer, 2013; Ferrer and Pardo, 2014) have compared, through a simulation study, the relative efficacies of the main methods. Application of all those methods requires some knowledge of the test properties, such as the population's mean and variance, or the reliability or internal consistency. However, these estimates are not available in some occasions. For example, sometimes it might not be possible to apply the complete test, but only a subset of its items. The properties of a test composed by that subset are therefore unknown. At other times the test is still in development. On other occasions the properties of the general population are known, but the test has been applied to an individual or group with special characteristics and it is uncertain whether the known properties of the test are generalizable to the subpopulation to which that individual or group belongs. In still others the test has been developed in a different country or in a different language and has not yet been adapted.

In short, sometimes we are interested in determining whether there has been a significant change in an individual's test score, taking as a basis the responses in the two applications, but we either do not know the characteristics of the test or we do not trust the ones we have available. Determining whether a measured change reaches significance can be based on the confidence intervals for the scores. Specifically, significant change is determined if the confidence intervals do not overlap. Our goal in this paper is to propose a procedure to create confidence intervals for an individual's scores without using any known psychometric properties of the test. Briefly, the procedure consists of performing a *bootstrap of items* (*BSI*), that is, in applying the bootstrap method to the responses given by an individual to the items of the test or scale.

## The bootstrap principle and psychometric measurement

The bootstrap method was originally developed to achieve statistical inference objectives while minimizing the risks of doubtful assumptions. The fundamental principle of the bootstrap is that if the sample is representative, then *it contains in some way the essential information of the population* (Efron, 1979; Efron and Tibshirani, 1993). In bootstrapping, a distribution of the statistic of interest is generated by means of intensive resampling with replacement of the values in the sample. That distribution is taken as an estimate of the statistic's sampling distribution. In contrast to conventional inference, no distributional assumptions are made, at least in the “nonparametric bootstrap” (Efron and Tibshirani, 1993). Samples of the same size as the original sample are taken from the distribution of observed values, with replacement. In each sample the statistic of interest is calculated and the distribution of the statistic's values obtained in this way is used as if it were its sampling distribution.

Congruent with the central idea behind the bootstrap, the procedure we propose (*BSI*) is to apply the bootstrap procedure to the sample of an individual's responses to the items. If the items that form the test adequately map the construct of interest we can assume that the individual's responses contain the essential information of that individual about the construct. That is, they contain the essentials of the responses that this individual would give to a hypothetical population of items that can measure the construct of interest. By resampling the responses to the items it is assumed that these responses allow us to obtain an approximation to the hypothetical distribution of applications of the test to that individual in the same conditions. While the bootstrap has been applied primarily to statistics such as the mean or the standard error, in *BSI* the statistic of interest is the score in the test (the sum of the values in the items).

In an ideal measurement we would use items containing the range of the construct exhaustively. Samples of these items would yield test values that oscillate around the true score. Since we cannot know the sampling distribution of the values in the test from the data of a specific individual, we obtain an approximate distribution by bootstrapping the available sample of responses to the items. In addition, a measurement error will occur in each test administration. Such measurement errors would also be reproduced and resampled to the extent that they are incorporated into the empirical responses of an individual to the items.

Applying *BSI* is simple; it is implemented by the following steps:

1. The responses given by the individual to the *J* items of the test or scale compose its *empirical distribution*.
2. Samples of *J* values of that distribution are extracted at random, with replacement, and the score in the test is calculated by summing each of these samples of responses. The score in the test with each sample is the statistic of interest.
3. After performing the above steps a large number of times (say, 3,000), a *sampling distribution* is constructed for the scores in the test.

The sampling distribution obtained is interpreted as an estimate of the distribution of scores that that individual would produce in a large number of administrations of similar tests, composed of items extracted from the same population of items when applied in those same conditions. That distribution can be used for different inferential objectives. For example, for the goal that has served as a starting point in this research: to obtain confidence intervals. For this, it is sufficient to obtain the corresponding percentiles in that distribution. If we apply the procedure above twice, one with the responses to the test before the intervention and the other with the responses after the intervention, we will have two confidence intervals that can be compared and checked for overlapping.

We assume that, because of the measurement error, if a test were applied (ideally) to the same individual an indefinitely large number of times, the scores that would be obtained would follow a certain distribution. Remember that the focus of our research is on the situation in which such a distribution is unknown. The classical approach is to approximate the distribution from estimates of some test properties and making additional assumptions. The *BSI* procedure allows us to obtain an approximation to this distribution when these properties are unknown and/or we do not want to make any distributional assumptions.

## Advantages of the *BSI* method

In principle, we are not proposing *BSI* as a preferable alternative to classical procedures, but rather as a resource for situations in which those procedures cannot be used. Even so, we find some advantages in our method over the classical procedures. First, application of the *BSI* requires no assumptions, so there is no risk of violating them. In classical procedures, the axioms and assumptions of classical test theory are usually in force (Gulliksen, 1950; Lord and Novick, 1968; Feldt and Brennan, 1989) as, for example, in the additive model (X = V + e), the assumption of independence is made (ρ_ve_ = 0) along with the assumption of a normal distribution of errors. Those axioms and assumptions could be incorrect, but this cannot happen with the *BSI*.

Second, unlike classical procedures, the *BSI* never needs to be adjusted because of inadequate values. When determining a confidence interval for the true score with the classical procedures some of its limits may be outside the range of possible scores in the test. In the distribution obtained by bootstrapping the smallest value will never be less than the lowest possible test score nor the largest will be greater than its maximum value. With *BSI* one never has to make such *ad hoc* adjustments. This problem simply cannot happen with *BSI*.

Third, with some traditional procedures the confidence interval has the same amplitude for all individuals, as the limits are obtained adding and subtracting the same amount to the individual's score: *z*·*Se* (the *z* value for the confidence level multiplied by the standard error of measurement). This is what happens in the classical procedure we use in the examples below (Gulliksen, 1950). On the contrary, with *BSI* the amplitude of the interval varies from one individual to another. This seems a desirable feature, since the uncertainty about the true score is not necessarily the same for all individuals. It is reasonable that the uncertainty is greater (and the interval is wider) when the responses to the items are heterogeneous than when they are homogeneous. Suppose, for example, a scale with Likert-type responses with 5 categories. An individual who responds mostly with values of 3 and 4 is very homogeneous in his/er values. On the other hand, another individual who responds with values throughout the scale could have the same total score on the test, but with more heterogeneous responses to the items. The uncertainty regarding the true score is greater in the second case, so that its confidence interval should have a greater amplitude.

## Assessing performance of *BSI* with some empirical examples

In order to evaluate the possible merits of the *BSI* procedure we have analyzed six databases. In each of them we have applied two procedures to detect the presence of significant differences; i.e., finding the confidence interval for *Estimating the True Score*, or *ETS* (Gulliksen, 1950), and applying the *BSI*. We have selected the *ETS* procedure above others (see other methods in Perdices, 2005; Ferrer and Pardo, 2014) because it is structurally most similar to the *BSI* method. In the first two databases we do not expect changes to occur in individuals. In fact we used a single administration of a test in which the items had been divided into two groups to form two half-tests that can be considered as parallel forms. In these cases we will assume that all individuals that meet the criterion for a significant change are false positives. The other four databases include groups in which reliable changes are expected to occur, since an intervention was performed between the two administrations of the test in order to produce a change in the characteristic measured. Therefore, the description of the first example will be the longest, since it includes the details of the division procedure in two halves and the calculation of the intervals by both procedures. The second example is very similar, so it will suffice to indicate the details of the test involved. The third explains the implications of expecting changes in a specific direction due to an intervention. The remaining three are very similar to the third, so only the particularities of the tests and samples will be indicated.

### NEOPI-R

We have analyzed separately the five main personality dimensions provided by the *NEOPI-R* (Costa and McCrae, 1992) in a sample of 179 participants. We do not have two administrations of the test, but rather the responses of a sample of individuals to a single application. We have divided the test of each dimension (48 items each) into two half-tests (24 items each), which we will consider as parallel forms. We assume that between the scores of an individual in both half-tests there is no real difference. Even so, we expect to observe a positive result (i.e., significant change) in a small proportion of individuals, corresponding to false positives. That proportion should equal the set alpha value. As in this case there is no expected change we have considered the differences in either of the two directions: any of the two half-tests larger than the other one. For this we have obtained bilateral confidence intervals.

The procedure consists in performing the following steps:

1. *Construction of parallel forms*. Two half-tests were generated by splitting the items so that the total scores in the two half-tests were as similar as possible. For this we have ordered the items according to the average score in the sample of 179 individuals, once recoded the inverse items through the *foreign* package (R Core Team, 2017). From the two items with the highest average score, the first was placed in the first half-test and the second in the second half-test. From the third and fourth items the third was assigned to the second half and the fourth to the first, and so on. Then the scores of the individuals in the two half-tests were calculated and significance tests applied to verify that neither the means nor the variances differed significantly. We found no significant differences in any of these tests. We consider that we have constructed two parallel forms administered at the same time, between which individuals should not have demonstrated any real change.
2. *ETS Confidence Interval*. To obtain the *ETS*, we first calculated the mean and variance of the empirical scores of the individuals in both half-tests, as well as the internal consistency of each half-test (Cronbach's alpha coefficient, obtained through the *psych R* package; Revelle, 2017). With these values we obtained the point estimate of the true score for each individual, *V*′, based on the score observed in that individual (*X*) and the statistics of the test ($\rho_{xx^{\prime }}$ and μ_*x*_ were estimated from the alpha coefficient and the means of the scores in the corresponding half-tests) (Gulliksen, 1950):
  $$V_{i}^{\prime }={\hat{\rho }}_{xx^{\prime }}\cdot X_{i}+\left(1-{\hat{\rho }}_{xx^{\prime }}\right)\cdot {\hat{\mu }}_{x}$$
  The limits of the 95% interval were obtained by adding to and subtracting from ${V_{i}}^{\prime }$ the estimate of the standard error of measurement multiplied by 1.96 (${\hat{\sigma }}_{x}$ is replaced by the standard deviation of the scores in the corresponding half-test):
  $${\hat{\sigma }}_{e}={\hat{\sigma }}_{x}\cdot sqrt\left(1-{\hat{\rho }}_{xx^{\prime }}\right)$$
  The results are limiting values of the *ETS* interval:
  $$95\%\;Cl:V_{i}^{\prime }\pm 1.96\cdot {\hat{\sigma }}_{e}$$
  To determine significant or reliable difference at the individual level, we found how many of the 179 participants show that the intervals of the half-tests do not overlap (the lower limit of one half-test is greater than the upper limit of the other).
3. *BSI Confidence Interval*. The *BSI* method was applied to each half-test of each individual, extracting samples of 24 responses from their items with replacement and calculating for each sample the score in that half-test (sum of the values in the 24 items extracted). After 3,000 repetitions the percentiles 2.5 and 97.5 were calculated for the distribution of scores of each half-test of each individual. For this purpose we have used the bias-corrected and accelerated method proposed by Efron and Tibshirani (1993) with the *bootstrap R* package (Tibshirani and Leisch, 2017). The presence of a reliable change was operationalized in the same way as in the previous method: determining how many individuals had non overlapping *BSI* intervals obtained in the two half-tests.

The results appear in Table 1. All the rates of significant change are lower than expected (5%). In none of the five dimensions did the rate achieved with either method exceed 2.8%. The reason for the small deviations with the *ETS* method is probably due to the fact that some of the assumptions are violated to some degree. More importantly, significant change rates with the *BSI* method are very similar. That is, a very similar result would have been achieved with the *BSI* method, despite not knowing (or using) the psychometric properties of the test.

**Table 1:** Frequencies (and percentages) of cases classified as showing no overlapping (significant change) in the examples analyzed (see the text), according to the *ETS* and *BSI* procedures.

**SCALE (N)**	***ETS* (%)**	***BSI* (%)**
*NEOPI-R* (*N* = 179)		
N	1 (0.6)	1 (0.6)
E	0 (0.0)	2 (1.1)
O	1 (0.6)	4 (2.2)
C	1 (0.6)	3 (1.7)
A	2 (1.1)	5 (2.8)
*STAI* (*N* = 417)	3 (0.7)	11 (2.6)
*Self-efficacy* (*N* = 110)	50 (45.5)	53 (48.2)
*CES-D* Group		
ACT (*N* = 33)	18 (54.5)	14 (42.4)
CBT (*N* = 30)	18 (54.5)	12 (40.0)
Control (*N* = 31)	4 (12.9)	3 (9.7)
*POMS* Group		
ACT (*N* = 33)	19 (57.6)	14 (42.4)
CBT (*N* = 28)	6 (21.4)	5 (17.9)
Control (*N* = 31)	4 (12.9)	5 (16.1)
*FMC-P* Group		
Cuba control (*N* = 98)	0 (0.0)	0 (0.0)
Spain control (*N* = 33)	2 (7.1)	2 (7.1)
Cuba experimental (*N* = 296)	186 (62.8)	156 (52.7)
Spain experimental (*N* = 93)	45 (48.4)	42 (45.2)

### State-trait anxiety inventory (STAI)

We have analyzed the responses of a sample of 417 individuals to a single administration of the *STAI* (Spielberger et al., 1982), a scale composed of 20 items. As in the previous example, we divided the test into two half-tests of 10 items with means and variances as close as possible. The procedures have also been the same. The results (Table 1) show similar rates of significant changes across individuals with both methods.

### Self-efficacy questionnaire

Panadero et al. (2012) assessed the effects of different types of self-assessment, instructions and feedback on self-regulated learning and self-efficacy. The tool employed to assess self-efficacy, composed of 8 items, was applied twice, before and after the intervention, to a sample of 118 participants. The final sample was composed of 110, as 8 participants had the same response to all eight items of the test before or after the intervention. As the distribution of tests scores obtained by bootstrap for those individuals is in fact a constant, it is not possible to obtain a confidence interval with the *BSI* method.

Both the *ETS* and *BSI* confidence intervals were obtained for each administration of the test in the same way as in the previous example with one exception. Since the expectation was that self-efficacy would increase as a consequence of the intervention, we classified as significant change those cases in which the lower limit of the post-intervention interval was higher than the upper limit of the pre-intervention interval. That is why the intervals were one-sided, calculated with *z* = 1.64 instead of 1.96 for the *ETS*. In *BSI* we calculated the 95th percentile of the pre-intervention distribution and the 5th percentile of the post-intervention distribution, instead of the 2.5 and 97.5th percentiles.

The results are also shown in Table 1. Again, the numbers of individuals showing significant changes are very similar, although with the *BSI* method the properties of the test have not been used.

### CES-D

Losada et al. (2015) assessed the impact of two interventions, Acceptance and Commitment Therapy (*ACT*; *N* = 33) and Cognitive-Behavioral Therapy (*CBT*; *N* = 30) for dementia family caregivers, and compared with a no-treatment control group (*N* = 31). The test employed was the Spanish version of the *CES-D* (*Center for Epidemiologic Studies-Depression* scale; Losada et al., 2012), composed of 20 items, and it was applied twice, before and after the intervention in the two first groups, and at the corresponding times for the control group.

Both the *ETS* and *BSI* confidence intervals were obtained for each administration of the test in the same way as in the previous example (one-side intervals). As the expectation was that the depressive symptomatology would decrease as a consequence of the interventions, we classified as showing significant change those cases in which the lower limit of the pre-intervention interval was higher than the upper limit of the post-intervention interval. We found one-sided intervals also for the control group, in order to make fair comparisons between the conditions.

The number of individuals showing significant change (Table 1) is slightly smaller with the *BSI* procedure than with *ETS* in the two treated groups, while they are very similar in the control group.

### POMS

The same groups as in the previous section were assessed for anxiety with the Tension-Anxiety subscale from the *POMS* scale (*Profile of Mood States*; McNair et al., 1971), composed of 8 items. It was applied together with the *CES-D* test described in the previous section, at the same time. Both the *ETS* and *BSI* confidence intervals were obtained for each administration of the test in the same way as in the previous example (one-sided intervals). As the expectation was that the anxiety measure would decrease as a consequence of the intervention, the procedures were exactly the same as with the *CES* example.

The numbers of individuals showing significant change (Table 1) is slightly smaller with the *BSI* procedure than with *ETS* for the *ACT* group, while they are very similar in the *CBT* and the control groups. It is worth to highlighting the fact that when one test (such as *POMS*) reflects a smaller effect than another test (such as *CES*) in a given group (the *CBT* group), this is reflected in the rates of significant change with both methods, *ETS* and *BSI*.

### Familiar motivational climate for parents (FMC-P)

Del Prado et al. (under review) assessed the effect of an intervention designed to improve the motivational climate for learning in families. Two groups (intervention and control) from two countries (Cuba and Spain) completed the scale before and after the intervention. The questionnaire employed, *FMC-P*, is composed of 14 variables (each one is the result of combining two questions). Table 1 shows that the rates of significant change are very close.

## Discussion

The procedure proposed, *bootstrap of items* (*BSI*), is a solution to the problem of assessing the presence of significant change when the properties of the test are not known (e.g., mean, variance, and reliability or internal consistency). In the *ETS* column in Table 1, the rates of significant change were obtained by employing the properties of the test. But our most striking result is that the rates in the *BSI* column are very similar to those for *ETS*, despite they have been obtained without knowing (or without using) any of those properties. We cannot state, with the evidence available so far, that *BSI* performs better than other procedures, such as *ETS*, nor recommend it when it is possible to use these other methods. However, in a situation such as we have considered in the present paper, when the properties of the test are not known or are not trustworthy, the *BSI* is a viable option to estimate confidence intervals.

Some studies have compared several of the indexes proposed in the literature to evaluate the presence of reliable change (Perdices, 2005; Ferrer and Pardo, 2014). Here we could have chosen any of them, other than *ETS*, to compare with the results of *BSI*. We know that these indices provide slightly different values. However, our interest here is not to determine what is best or which provides results more similar to those obtained with *BSI*. Our interest is mainly to show that the *BSI* procedure provides useful results, with rates of significant change in an acceptable range, similar to those provided by a classical and structurally-similar procedure such as *ETS*.

The fundamental principle of the method is the same as in other contexts in which bootstrapping is applied (Efron, 1979; Efron and Tibshirani, 1993): if the test contains a reasonably large number of items and these properly represent the construct being measured, then they contain enough essential information to approximate a hypothetical distribution of person's scores in order to make inferences at the individual level. In the present paper we have formulated the fundamental principle of the *BSI* and have shown that it yields promising results.

The *BSI* procedure has some undoubted merits. The main one is that it provides a solution when other methods cannot be applied. It is also important to emphasize that its application does not depend on assumptions that can be somewhat risky. Even in situations in which the psychometric properties of the test are known, it could be advisable to use *BSI* instead of other procedures that involve more assumptions. In fact, the immediate temptation when assessing how well *BSI* performs is to compare its results with those of other procedures such as *ETS*. However, in any application with real data we cannot be sure that a discrepancy between its results and those of other procedures has occurred because the *BSI* performs is inferior. Let us consider the examples with interventions that we have discussed above. In some of them we have found such discrepancies. But the *ETS* procedure is based on certain assumptions that we cannot independently verify (i.e., the assumption of normality). It is possible that any violations of these assumptions leads to inappropriate rates of non-overlapping *ETSs* intervals across individuals. It is possible that the rates provided by *BSI* are actually more precise.

However, *BSI* also has some drawbacks. When an individual responds very homogeneously to the test items, the *BSI* interval can be too narrow. In an extreme case in which responses to all the items are the same, the values obtained by bootstrap for the statistic are a constant. In such cases *BSI* is not suitable. How much variability in the items responses is required for the *BSI* interval to perform properly? It is necessary to initiate a research program that answers that question and other multiple questions that might arise regarding its practical implications. These include: (1) How many items are necessary for the *BSI* to perform validly and efficiently? (2) Is the response format relevant, and, for example, how many alternatives should be used in a Likert scale? (3) How many cycles of resampling are necessary for the distribution to be stable? (4) Since in our analyses the *BSI* has shown to be somewhat conservative, will it work better with nominal rates of somewhat higher false positives (alpha), such as 10%?

The *BSI* procedure also has potential applications when combined with other methodologies, such as meta-analysis. Specifically, in the meta-analysis of individual participant data (Debray et al., 2015) measurements of the different individuals are sometimes not directly comparable. The *BSI* opens new possibilities in the development of this type of meta-analysis.

## Author contributions

JB: Study conception, methodology, formal analysis, resources, writing/manuscript preparation of the initial draft. DB: Methodology, computation, resources, writing/manuscript preparation, critical review. MS: Study conception, formal analysis, writing/manuscript preparation, critical review. JJ, Study conception, writing/manuscript preparation, critical review.

### Conflict of interest statement

The authors declare that the research was conducted in the absence of any commercial or financial relationships that could be construed as a potential conflict of interest.
